# Primary costal hydatid cyst resembling urolithiasis: A case report

**DOI:** 10.1016/j.ijscr.2023.107888

**Published:** 2023-01-12

**Authors:** Ershadi Reza, Amini Hesam, Soltanmohammadi Sara, Issaiy Mahbod, Rafieian Shahab

**Affiliations:** a Department of Thoracic Surgery, Imam Khomeini Hospital Complex, Tehran University of Medical Sciences, Tehran, Iran; b Department of Pulmonology, Imam Khomeini Hospital Complex, Tehran University of Medical Sciences, Tehran, Iran; c School of Medicine, Tehran University of Medical Sciences, Tehran, Iran

**Keywords:** Hydatid cyst, echinococcus granulosus, Costal hydatid disease, Thoracotomy

## Abstract

**Introduction:**

Hydatid disease (HD) is a zoonotic infection caused by echinococcus granulosus tapeworms. HD accounts for approximately one million cases worldwide. HD is more prevalent in endemic areas, such as the Mediterranean region.

**Presentation of case:**

A middle-aged male patient presented with right flank pain for years. His vital signs and physical examination were unremarkable. The whole-body bone scan revealed an area along the sixth rib's posterior arch with increased metabolic activity and CT and MRI were compatible with a hydatid cyst. The cyst was surgically resected and irrigation of the area with hypertonic saline was done. Medical treatment with albendazole was initiated and the patient had no complications.

**Discussion:**

Bone involvement is an uncommon finding in HD and involvement of the ribs is even more scarce. Surgical resection complemented with medical treatment is the preferred approach.

**Conclusion:**

In the Mediterranean and middle eastern regions, high infection rates with E. granulosis are evident hence, uncommon manifestations of the disease should be regarded. Although renal stones are far more prevalent when in endemic areas of HD, the physician should also consider skeletal HD as a differential diagnosis.

## Highlights

• Bone involvement is an uncommon finding in hydatid disease and involvement of the ribs is even more scarce.
• A middle-aged male patient with a past medical history of renal stones was referred.
• The whole-body bone scan revealed an area along the sixth rib posterior arch.
• Imaging revealed a cystic lesion with a daughter cyst measuring 46 mm in total and located at the sixth rib's posterior arch.
• The treatment of choice would be surgery, complemented with albendazole.

## 1 Introduction

Hydatid disease (HD) is a zoonotic infection caused by echinococcus granulosus tapeworms. Humans act as accidental intermediate hosts for the parasite. They usually get infected by ingesting contaminated water, food, or soil [1]. The frequency of HD is more than one million worldwide. However, it is more prevalent in endemic areas, particularly in the Mediterranean region. The annual incidence rate is 50 per 100,000 people and the prevalence might exceed 5 %–10 % in the endemic areas [2]. In Iran, an endemic country, the prevalence of Cystic Echinococcosis (CE) is 5 %. HD might be asymptomatic, specifically when bones are involved [3]. Rib involvement usually mimics other diseases. Therefore, physicians might misdiagnose it as a chest wall tumor [4], outlet thoracic syndrome (when the 1st rib is affected), or Pancoast tumor (in apical involvement), etc. In this report, we present a rare case of HD with costal involvement. This work has been reported in line with the SCARE criteria [5].

## 2 Case presentation

**Fig. 1:**
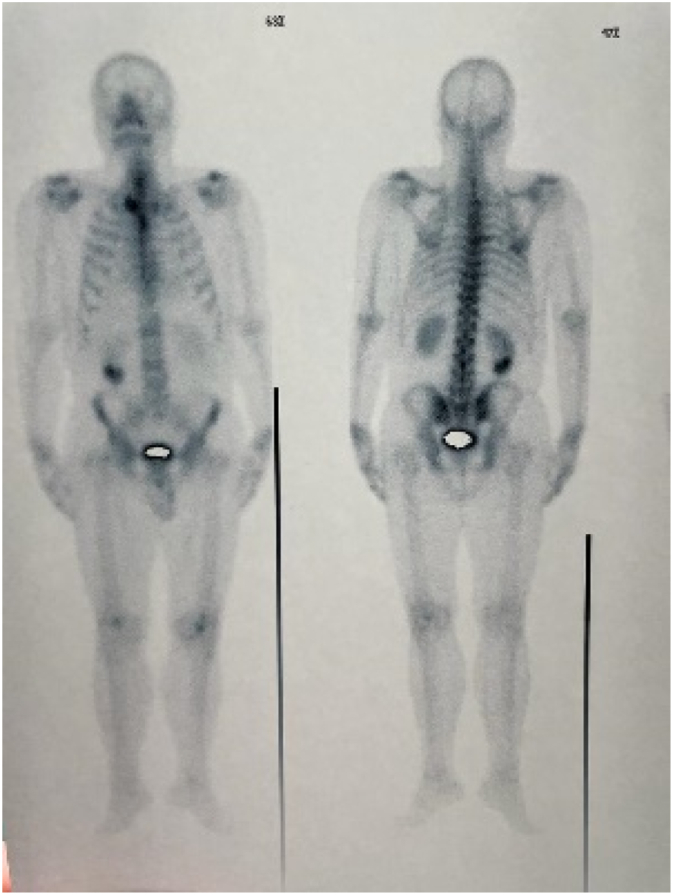
Anterior and posterior views of planar bone scan showing increased activity in the sixth rib on the right side.

**Fig. 2:**
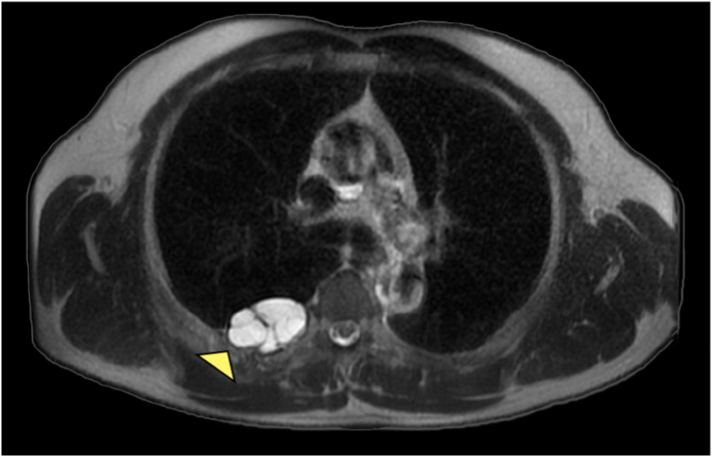
Axial T2-weighted MRI showing a cystic lesion with a daughter cyst measuring 46 mm in total located at the sixth rib's posterior arch.

**Fig. 3:**
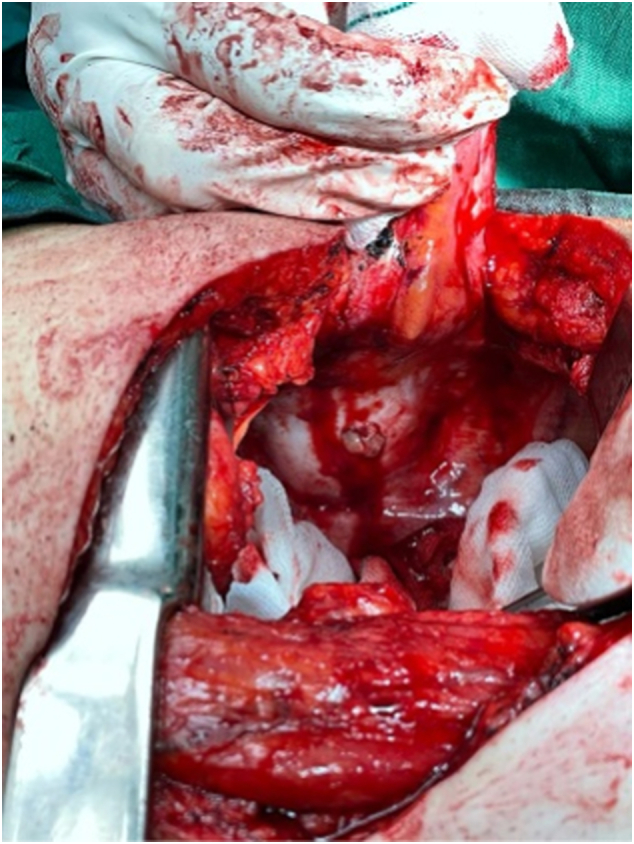
Thoracotomy incision, hydatid cyst is visible.

**Fig. 4:**
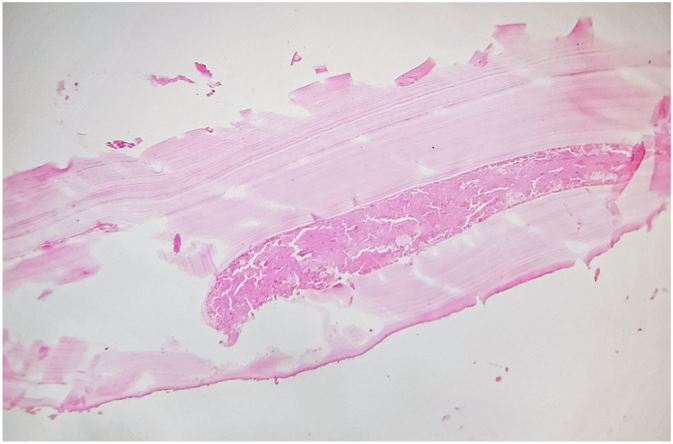
Photomicrograph showing acellular lamellar calcification (H&E stain, ×40).

**Fig. 5:**
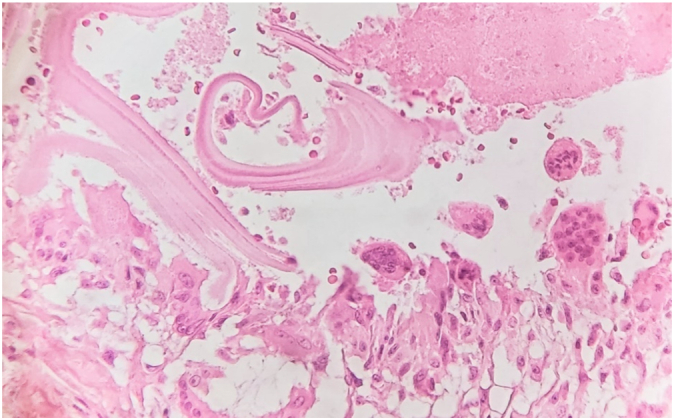
Photomicrograph showing acellular lamellar calcification with multinucleated giant cells and histiocytic reaction (H&E stain,×40).

A 56-year-old middle eastern male patient was referred to the thoracic surgery clinic with right-sided flank pain and an eight-kilogram weight loss in four months. He had been suffering from symptoms resembling renal colic for fifteen years. The patient had undergone multiple surgical interventions for nephrolithiasis and the current episode of pain was located on the same side as the previous renal colic. Furthermore, he was diagnosed with hypertension and was under medical treatment with amlodipine and valsartan. He was smoker and had no history of substance abuse. His initial vital signs were all within normal ranges: blood pressure 115/80 mmHg, pulse rate 87 bpm, respiratory rate 15 pm, temperature 37.2 °C, oxygen saturation 96 %. physical examination was unremarkable and no local tenderness was detected. The bone scan disclosed a hypermetabolic bone involvement in the sixth rib on the right side (e.g., inflammatory process, bone metastasis, etc.) (Fig. 1). He was admitted to the thoracic surgery ward for further investigations. Chest computed tomography without contrast and magnetic resonance imaging studies revealed a cystic lesion with a daughter cyst measuring 46 mm in total located at the sixth rib's posterior arch (Fig. 2). The lesion had significantly deformed the surrounding bone and had led to osteolysis. No pleural effusion or pulmonary lesion was detected. The radiological appearance was compatible with a hydatid cyst. Thoracoscopic surgery was planned for diagnosis and treatment Thus, Single port thoracoscopy was performed. The intraoperative finding was an intraosseous cystic lesion measuring about 6 cm in the extrapleural space located at the posterior arch of the sixth rib. For better exposure and more explorative capabilities, the procedure was converted from thoracoscopy to open surgery. A right posterolateral incision through the 5th intercostal space was made. The site was irrigated with hypertonic saline as scolicidal agent. Thereafter, the cyst was incised, and the membranes were removed. Finally, partial resection of the sixth rib was undertaken. The seventh rib at the posterior angle was also involved and resection of the involved part of the rib was also done. A chest tube and a feeding tube for leaving the pleural space were placed in the cyst cavity (Fig. 3). Histopathologic evaluation of the mass revealed “Hydatid cyst with prominent foreign body type granulomatous inflammation at the periphery between skeletal muscles with extension to osteocartilaginous tissue.” (Fig. 4, Fig. 5).

Postoperative treatment included albendazole tablets 400 mg twice daily. The patient had no respiratory complications, and the chest tube was removed on the fifth-day post-operation. The chest X-ray findings on the discharge were unremarkable. The patient was reevaluated four weeks after the surgery, and he had no major complications. He only complained of slight somatic pain at the incision site. Chest X-ray was normal, and the wound was healed. The patient is currently being monitored with radiological surveillance every 3 months.

## 3 Discussion

HD is a zoonotic infection that occurs both in animals and humans. The two common types of echinococcosis that infect humans the most include *E. granulosus* (cystic HD) and E. multilocularis (alveolar HD) and the former is more prevalent [6]. HD may occur in any part of the body; however, the most common sites are the liver (59–75 %) and the lungs (27 %). Bone involvement is an uncommon finding in HD (only in 1–4 % of the cases [7]) and the spine is afflicted in almost half of them [8]. Hydatid cyst of the ribs is even more scarce. Clinical symptoms of HD are related to the location and the size of the involvement and small cysts can remain asymptomatic for long periods [9]. Chest wall cysts can resemble tumors and lead to misdiagnosis [10]. CE can be diagnosed based on clinical, imaging, and serologic findings [11]. However, in the case of costal HD, radiologic findings might be more helpful. The treatment of choice would be radical excision of the lesion with a margin [12]. In addition to surgical resection, medical treatment with albendazole is also required for maximum efficacy [13].

## 4 Conclusion

Hydatid cyst of the rib is a remarkably rare condition. Rib hydatid cysts might be misdiagnosed with renal stones or costal tumors due to their shared signs and symptoms. In endemic regions, however, hydatid cysts should be considered in the differential diagnosis whenever the symptoms are suggestive. The treatment of choice would be surgery, complemented with albendazole.

## Sources of funding

N/A.

## Consent

Written informed consent was obtained from the patient for publication of this case report and accompanying images. A copy of the written consent is available for review by the Editor-in-Chief of this journal on request.

## Ethical approval

This case report does not hold any personal information leading to the identification of the patient. Therefore, it is exempted from ethical approval.

## CRediT authorship contribution statement

Amini H: Conceptualization, Writing Original- Draft, Investigation, Resources.

Issaiy M: Methodology, Validation, Supervision, Writing- Review and editing.

Rafieian Sh, Soltanmohammadi S, and Ershadi R: Writing Original- Draft, Resources.

## Declaration of competing interest

N/A.
